# Supplementary material for: Nigrostriatal dopamine pathway regulates auditory discrimination behavior
Source: Nat Commun. 2022 Oct 8;13:5942. doi: 10.1038/s41467-022-33747-2 (PMC9547888; doi:10.1038/s41467-022-33747-2)
Supplement: Supplementary file 1 — Supplementary Information [file 41467_2022_33747_MOESM1_ESM.docx]

**
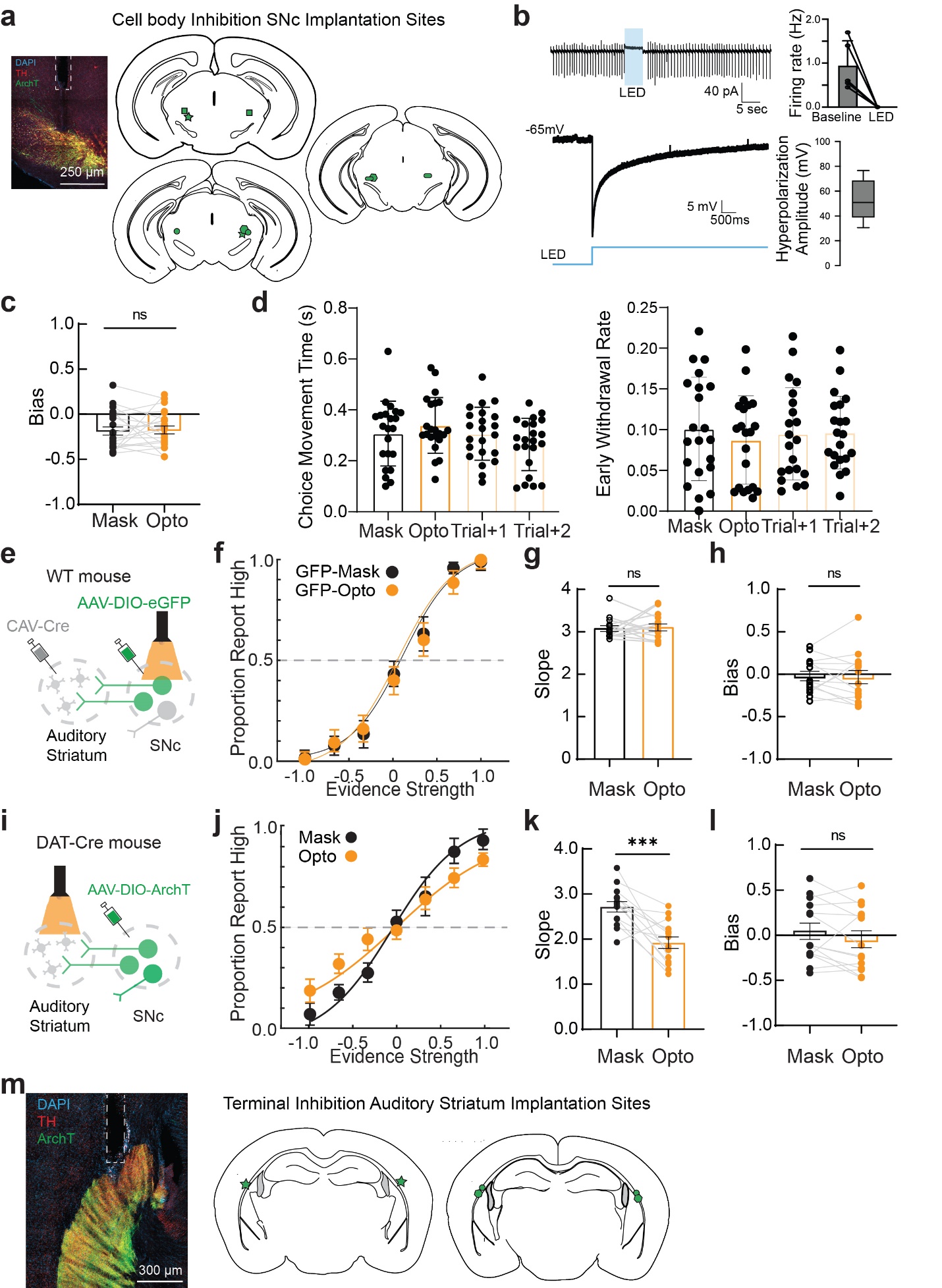
Supplementary Figure 1. Summary of optogenetic experiment histology and associated control experiments.** (**a**) Left, representative fiber placement for substantia nigra pars compacta (SNc) cell body inhibition. Right, summary of bilateral fiber placements. Each green shape represents a different experimental animal included in the analysis for **Fig. 1e-j**. (**b**) Upper left, example cell attached voltage recording from an ArchT-GFP -positive SNc neuron showing tonic firing at rest and transient silencing in response to an LED pulse (blue box). Upper right, quantification of firing suppression from all recorded neurons (n = 5). Lower left, example whole cell current clamp recording from an ArchT-GFP -positive SNc neuron showing a robust hyperpolarization in response to an LED pulse (blue line). Lower right, quantification of hyperpolarization amplitude from all 5 recorded neurons. Error bars are SEM. Box plot is Tukey format: bottom whisker represents the minimum, bottom bound is the 25^th^ percentile, median is center, top bound is 75^th^ percentile, and top whisker represents maximum. (**c**) Comparison of corresponding bias indices for individual sessions. Bars represent the mean, and dots represent the individual sessions (n.s. p = 0.19, two-sided Wilcoxon rank-sum test). Black indicates masking light trials, and orange indicates optical inhibition trials (n = 21 sessions across 5 mice). (**d**) Left, effects on mean choice movement time. Right, effects of optogenetic inhibition on early withdrawal rate. Data are presented as the mean ± S.D. (n.s, p > 0.05, two-sided paired t-test). Individual dots represent individual session values. Data are mean ± SEM. (**e**) Schematic for optogenetic silencing using fluorophore expression (eGFP instead of ArchT-GFP) as a control for Figure 1. (**f**) Psychometric performance during stimulus-based silencing comparing optogenetic light vs. masking light only. Data per evidence strength are presented as the mean ± SEM. (n = 3 mice across 15 sessions). (**g**) Comparison of corresponding regression slopes for individual sessions contributing to the analysis in **f**. Bars represent the mean and dots represent individual sessions (n.s. p = 0.98, two-sided Wilcoxon rank-sum test). (**h**) Comparison of corresponding bias indices for individual sessions. Bars represent the mean, and dots represent individual sessions (n = 15 sessions across 3 mice; n.s. p = 0.54, two-sided Wilcoxon rank-sum test). Data are mean ± SD. (**i**) Schematic for terminal optogenetic silencing. (**j**) Psychometric performance during stimulus-based silencing comparing optogenetic light vs. masking light only. Data per evidence strength are presented as the mean ± SEM. (n = 3 mice across 14 sessions). (**k**) Comparison of corresponding regression slopes for individual sessions contributing to the analysis in J. Bars represent the mean and dots represent individual sessions (n = 3 mice across 14 sessions; ***p = 0.002, two-sided Wilcoxon rank-sum test). Data are mean ± SEM. (**l**) Comparison of corresponding bias indices for individual sessions. Bars represent the mean, and dots represent individual sessions (n = 3 mice across 14 sessions; n.s., two-sided Wilcoxon rank-sum test). Data are mean ± SEM. (**m**) Summary of bilateral fiber placements for the optogenetic terminal inhibition experiments shown in Supplementary **Fig.1i-l**. Each green shape represents a different experimental animal included in the analysis.


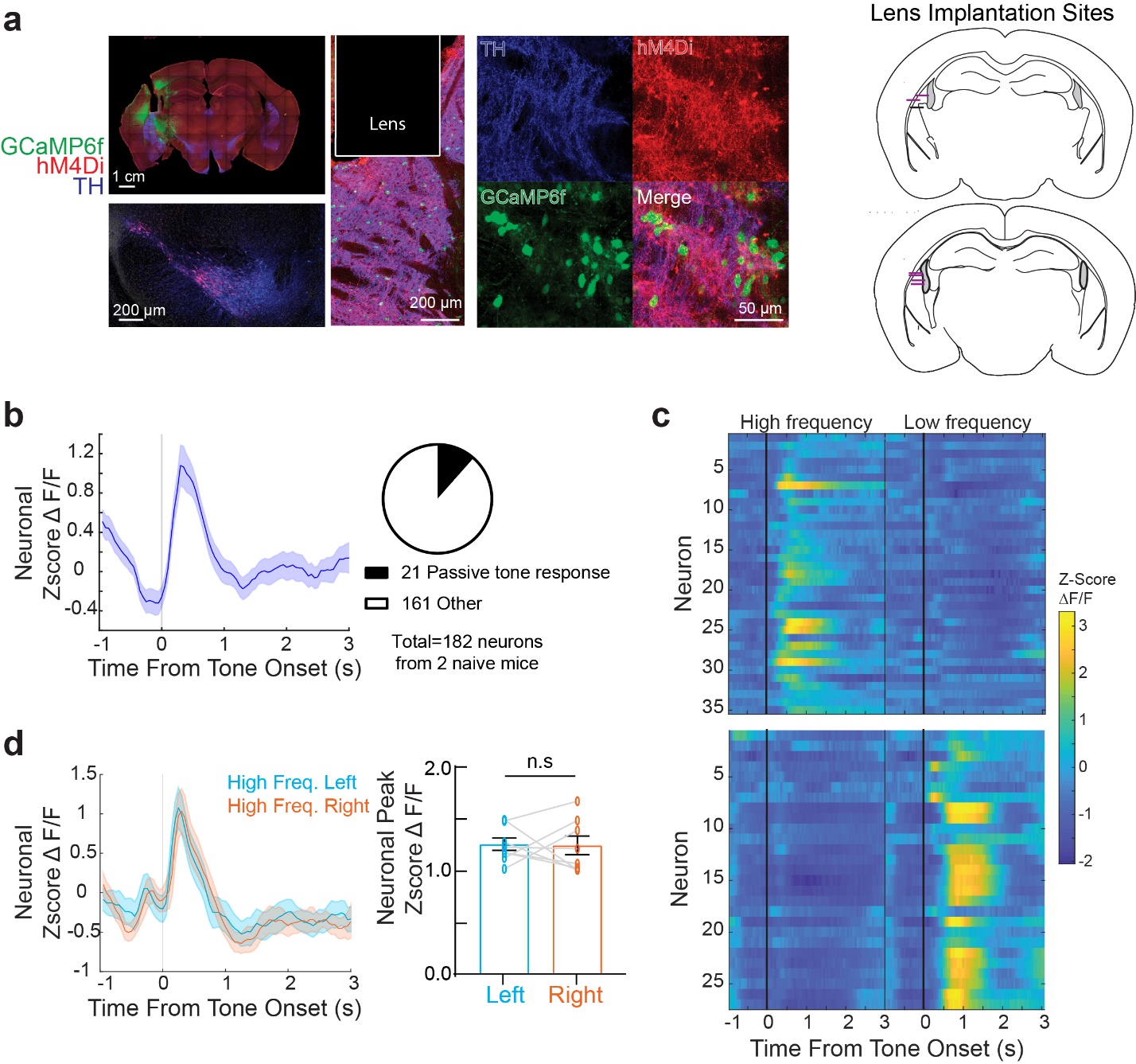


**Supplementary Figure 2. Auditory striatal microendoscopic imaging and chemogenetic silencing.** (**a**) Representative histology for simultaneous auditory striatal calcium imaging and chemogenetic substantia nigra pars compacta (SNc) silencing. Left top, representative lens placement for auditory striatal calcium imaging. Left bottom, expression of hM4Di-mCherry in the SNc with tyrosine hydroxylase (TH, blue) visualization. Middle, 20× and 40× confocal images of lens placement sites. Co-expression of hM4Di-mCherry (red) and TH (blue) in auditory striatal terminals with neurons labeled with GCaMP6f (green). Right, summary of lens implantation sites for simultaneous dopamine sensor imaging and chemogenetic SNc silencing. Purple lines represent the bottoms of the lenses implanted in each animal (n = 7 mice). (**b**) Striatal tone responses in naïve mice. Left, tone-evoked response from one example neuron. Right, quantification from all neurons recorded in naïve mice (n.s., p > 0.05, Mann–Whitney U test). Data are mean ± SEM. (**c**) Example auditory striatal neurons with tone frequency preferences: high-frequency preferred neurons (top) and low-frequency preferred neurons (bottom). (**d**) Example neurons showing response towards the same stimuli in correct and error trials. Left, one example neuron. Right, quantification from the eight neurons (6 sessions, 142 trials). Data are mean ± SEM for both left and right panels.

**
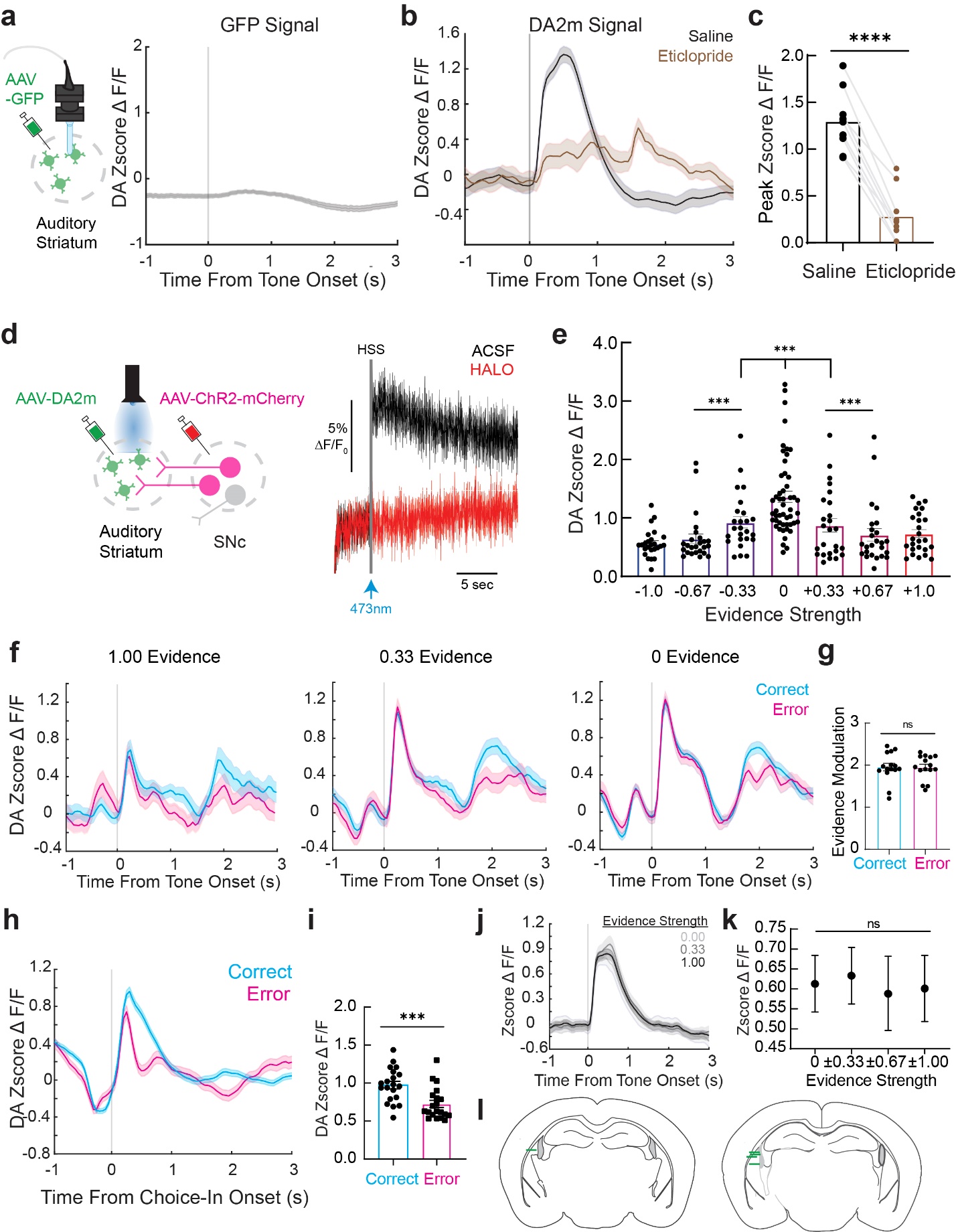
**

**Supplementary Figure 3. Validation and behavioral correlates of microendoscopic dopamine measurement in the auditory striatum.** (**a**) Left, schematic for recording GFP control signals during the performance of an auditory discrimination task. Right, averaged tone-aligned traces for GFP imaging during task performance. (**b**) Averaged traces of tone-evoked dopamine responses following saline (black) or D2R antagonist eticlopride (brown) i.p injection (0.1 mg/kg). Error bars are SEM. (**c**) Peak dopamine responses following either saline (black) or eticlopride (brown) treatment sessions. Data are presented as the mean per session (****p < 0.0001, two-sided Mann–Whitney U test). (**d**) Left, schematic for two-photon optogenetic *ex vivo* slice validation of dopamine sensor function in the auditory striatum. Right, averaged DA2m fluorescence tracing during the optical activation of nigrostriatal axons in the auditory striatum of slices bathed in artificial cerebrospinal fluid (aCSF; Black) or aCSF with haloperidol (Red). (**e**) Unfolded psychometric dopamine responses as a function of evidence strength. (***p < 0.001, two-sided Wilcoxon-rank sum test). Individual dots represent averaged traces per session as in Figure 3E. Data are mean ± SEM. (**f**) Tone-evoked DA responses between correct and error trials. Left, tonal responses for evidence strength 1.0 (n = 3 animals; 12 sessions, 82 error trials and 82 trials randomly selected from 950 correct trials). Middle, tonal responses for 0.33 evidence strength (n = 3 animals, 8 sessions, 281 error trials and 281 trials randomly selected from 462 correct trials). Right, tonal responses for 0 evidence strength (n = 3 animals, 10 sessions, 309 for error and 309 for correct trials). Error bars are SEM. (**g**) Evidence modulation as a function of correct and incorrect trials (n.s., p > 0.05, two-sided Wilcoxon rank-sum test). Each individual dot represents an individual session’s calculation sorted by either correct or incorrect trials. Data are mean ± SEM. (**h**) Averaged dopamine responses aligned with the port response choice timestamp for correct and incorrect trials. Error bars are SEM. (**i**) Quantification of peak dopamine responses relative to the port response choice timestamp for correct and incorrect trials (^***^p =0.0007, two-sided Wilcoxon rank-sum test). Each individual dot represents averaged peak values per session sorted by either correct or incorrect trials (n = 20 sessions across 5 mice). Data are mean ± SEM (**j**) Passive tone-evoked dopamine responses to stimuli containing different levels of evidence. (**k**) Passive peak responses following the presentation of stimuli containing different levels of evidence (ns, p < 0.05, Wilcoxon rank-sum test). For averaged trace and quantification data, data are presented as the mean ± SEM (n = 20 sessions across 5 mice). (**l**) Summary of lens implantation sites for experiments shown in **Fig. 3a-g** (n = 5 mice). Each green line represents a different experimental animal included in the analysis for **Fig. 3**.


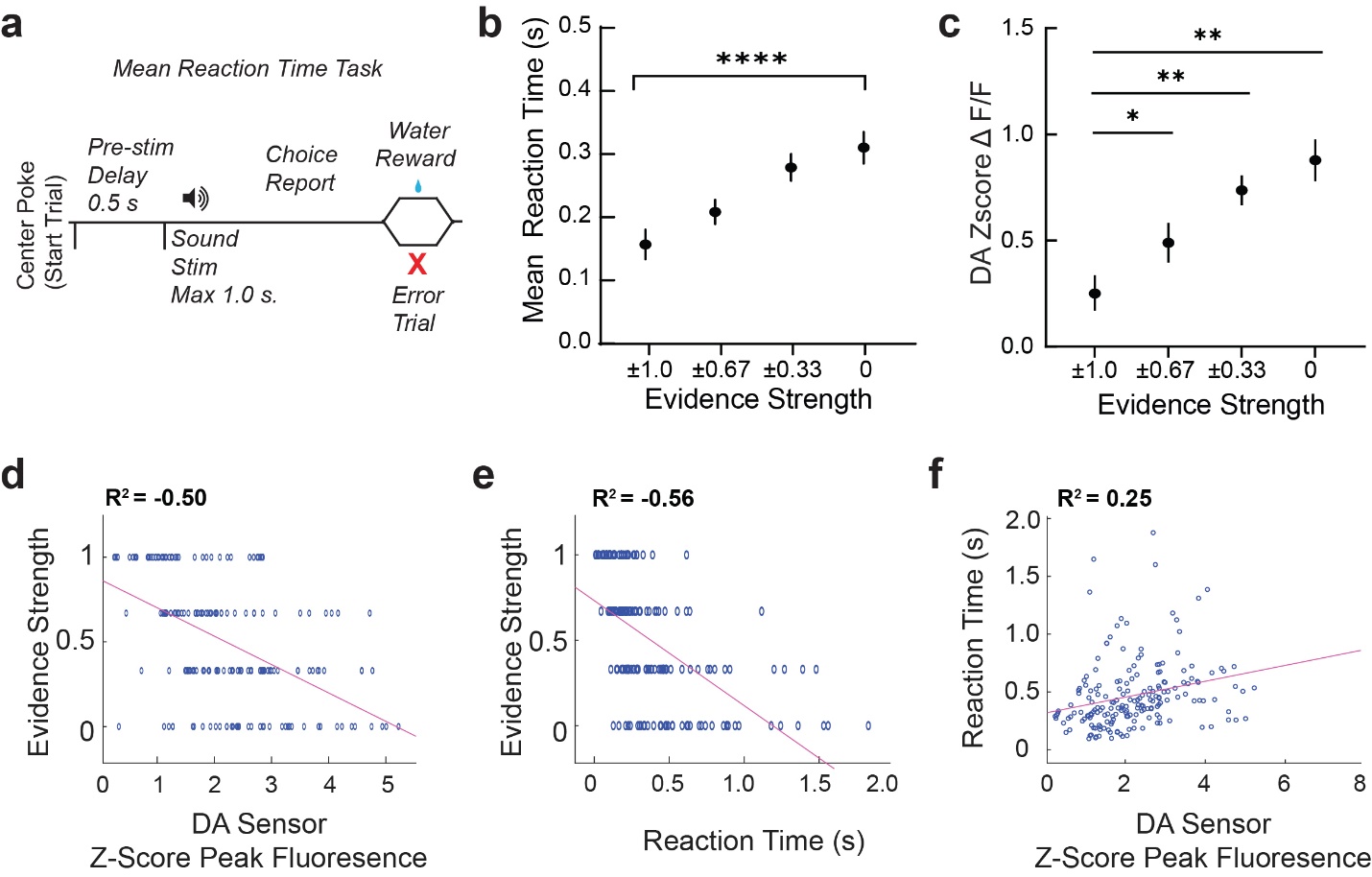


**Supplementary Figure 4. Correlations among evidence strengh, striatal dopamine activity, and reaction time.** (**a**) Reaction time task sequence with concurrent DA2m recordings in mice. (**b**) Mean reaction times as a function of evidence strength (^****^ *p < 0.0001,* unpaired Wilcoxon rank-sum test). (**c**) Peak DA2m dopamine responses during reaction time tasks, as a function of evidence strength (^**^p = 0.0032 for 0.33 to 1.00 evidence strength comparison, ^**^p = 0.0090 for 0.00 to 1.00 evidence strength comparison, ^*^p = 0.03 for 1.00 to 0.67 evidence strength comparison; unpaired Wilcoxon rank-sum test). (**d**) Correlation plot and R^2^ between evidence strength and the same trial tone-evoked DA sensor peak responses. (**e**) Correlation plot and R^2^ between evidence strength and same-trial reaction time. (**f**) Correlation plot and R^2^ between reaction time and the same trial tone-evoked DA sensor peak responses. Blue dots are individual trials and the red line is the correlation regression. In **b** & **c**, Data are presented as the mean ± SEM. *p < 0.05, **p < 0.01, ***p < 0.001, ****p < 0.0001, one-way ANOVA. n = 3 mice, 6 sessions, 191 trials.

**
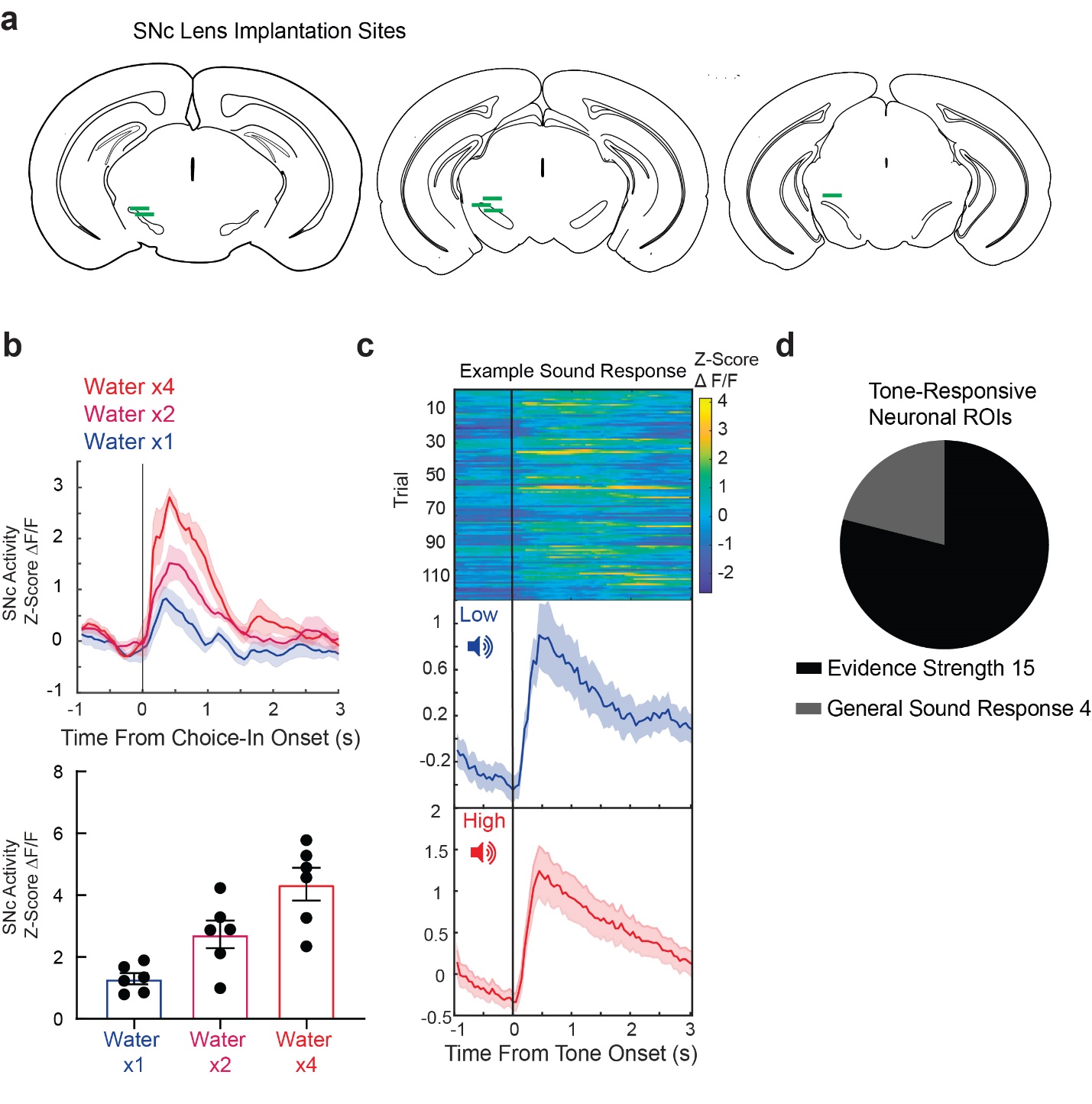
**

**Supplementary Figure 5. CAV-mediated calcium imaging of auditory striatal–projecting SNc dopamine neurons.** (**a**) Summary of lens placement histology for substantia nigra pars compacta (SNc) recordings using combined canine adenovirus 2 (CAV)-Cre and GCaMP6f infusion. Green represents the lens bottom for each animal (n = 6 mice). (**b**) SNc neurons that respond to changes in reward magnitudes (n = 6 neuronal ROIs). Data are mean ± SEM for both top and bottom panels. (**c**) Representative neuron that is responsive to sound during a single task session. Blue represents low frequency, and red represents high frequency. Data are presented as the mean ± SEM. (**d**) Proportions of tone-responsive neurons modulated by evidence strength.


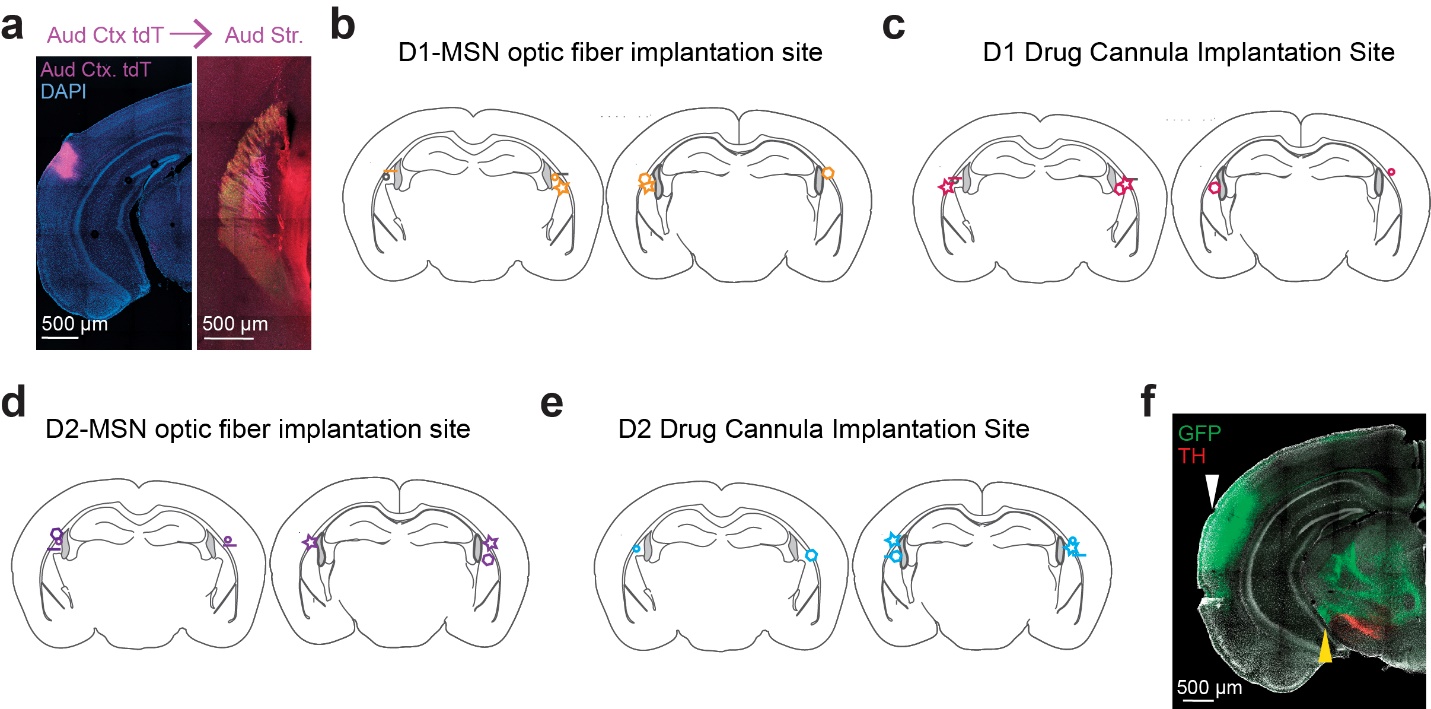


**Supplementary Figure 6. Additional anatomical characterization.** (**a**) Anterograde tracing from the auditory cortex (tdT fluorescence in magenta to the auditory striatum). (**b**) Summary of bilateral optic implantation sites for D1-MSN optogenetic silencing experiments using ArchT. Orange objects represent fiber tip placements in each animal. (**c**) Summary of bilateral cannular implantation sites for D1 receptor antagonist SCH-23390 infusion experiments. Red objects represent cannula tips in each animal. (**d**) Summary of bilateral optic implantation sites for D2-MSN optogenetic silencing experiments using ArchT. Purple objects represent fiber tip placements in each animal. (**e**) Summary of bilateral cannular implantation sites for D2 receptor antagonist Sulpiride infusion experiments. Cyan objects represent cannula tip placements in each animal (**f**) Anterograde tracing of the primary auditory cortex. The white arrow indicates the injection site, and the yellow arrow indicates terminal projections to the lateral substantia nigra.
