# Supplementary material for: Nigrostriatal dopamine pathway regulates auditory discrimination behavior
Source: Nat Commun. 2022 Oct 8;13:5942. doi: 10.1038/s41467-022-33747-2 (PMC9547888; doi:10.1038/s41467-022-33747-2)
Supplement: Supplementary file 3 — Description of Additional Supplementary Files [file 41467_2022_33747_MOESM3_ESM.docx]

**Description of Additional Supplementary Files**

**Supplementary Movie 1. Example video for DA sensor (DA2m) imaging in the auditory striatum.** The overall fluorescent intensity changes of DA sensor in the auditory striatum under the imaging field.
