# Supplementary material for: Nigrostriatal dopamine pathway regulates auditory discrimination behavior
Source: Nat Commun. 2022 Oct 8;13:5942. doi: 10.1038/s41467-022-33747-2 (PMC9547888; doi:10.1038/s41467-022-33747-2)
Supplement: Supplementary file 5 — Reporting Summary [file 41467_2022_33747_MOESM5_ESM.pdf]

nature portfolio

Corresponding author(s): Qiaojie Xiong

Last updated by author(s): Sep 15, 2022

Reporting Summary

Nature Portfolio wishes to improve the reproducibility of the work that we publish. This form provides structure for consistency and transparency in reporting. For further information on Nature Portfolio policies, see our Editorial Policies and the Editorial Policy Checklist.

Statistics

For all statistical analyses, confirm that the following items are present in the figure legend, table legend, main text, or Methods section.

n/a	Confirmed	
☐	☒	The exact sample size (n) for each experimental group/condition, given as a discrete number and unit of measurement
☐	☒	A statement on whether measurements were taken from distinct samples or whether the same sample was measured repeatedly
☒	☐	The statistical test(s) used AND whether they are one- or two-sided<br>*Only common tests should be described solely by name; describe more complex techniques in the Methods section.*
☐	☒	A description of all covariates tested
☐	☒	A description of any assumptions or corrections, such as tests of normality and adjustment for multiple comparisons
☐	☒	A full description of the statistical parameters including central tendency (e.g. means) or other basic estimates (e.g. regression coefficient) AND variation (e.g. standard deviation) or associated estimates of uncertainty (e.g. confidence intervals)
☐	☒	For null hypothesis testing, the test statistic (e.g. F, t, r) with confidence intervals, effect sizes, degrees of freedom and P value noted<br>*Give P values as exact values whenever suitable.*
☒	☐	For Bayesian analysis, information on the choice of priors and Markov chain Monte Carlo settings
☒	☐	For hierarchical and complex designs, identification of the appropriate level for tests and full reporting of outcomes
☒	☐	Estimates of effect sizes (e.g. Cohen's d, Pearson's r), indicating how they were calculated

Our web collection on statistics for biologists contains articles on many of the points above.

Software and code

Policy information about availability of computer code

Data collection	Behavior monitoring and measurements were obtained using Bpod state machine rl (Sanworks,R ochester,U SA). Behavioral data were measured and acquired using custom-written scripts in MATLAB R2014a (The Mathworks,In c.,N atick,M assachusetts,U SA). Data acquisition for microendoscopic recordings was performed using nVista Data Acquisition Software (Inscopix,P alo Alto,C A).
Data analysis	Behavioral data and associated parameters were analyzed using custom written scripts in MATLAB R2020b (The Mathworks,In c.,N atick, Massachusetts,U SA). Microendoscopic data processing was performed using Mosaic (Inscopix,P alo Alto,C A). Cellular extraction and further data analysis was performed by using publicly available CNMF-E and custom written scripts in MATLAB. Statistical analyses were performed in Graphpad Prism 8 {Graphpad Software Inc.).

For manuscripts utilizing custom algorithms or software that are central to the research but not yet described in published literature, software must be made available to editors and reviewers. We strongly encourage code deposition in a community repository (e.g. GitHub). See the Nature Portfolio guidelines for submitting code & software for further information.

Data

Policy information about availability of data

All manuscripts must include a data availability statement. This statement should provide the following information, where applicable:

- Accession codes, unique identifiers, or web links for publicly available datasets
- A description of any restrictions on data availability
- For clinical datasets or third party data, please ensure that the statement adheres to our policy

All data are provided in the main text or supplementary data. Custom written codes and raw data are available upon reasonable request.

Human research participants

Policy information about studies involving human research participants and Sex and Gender in Research.

Reporting on sex and gender	*Use the terms sex (biological attribute) and gender (shaped by social and cultural circumstances) carefully in order to avoid confusing both terms. Indicate if findings apply to only one sex or gender; describe whether sex and gender were considered in study design whether sex and/or gender was determined based on self-reporting or assigned and methods used. Provide in the source data disaggregated sex and gender data where this information has been collected, and consent has been obtained for sharing of individual-level data; provide overall numbers in this Reporting Summary. Please state if this information has not been collected. Report sex- and gender-based analyses where performed, justify reasons for lack of sex- and gender-based analysis.*
Population characteristics	*Describe the covariate-relevant population characteristics of the human research participants (e.g. age, genotypic information, past and current diagnosis and treatment categories). If you filled out the behavioural & social sciences study design questions and have nothing to add here, write "See above."*
Recruitment	*Describe how participants were recruited. Outline any potential self-selection bias or other biases that may be present and how these are likely to impact results.*
Ethics oversight	*Identify the organization(s) that approved the study protocol.*

Note that full information on the approval of the study protocol must also be provided in the manuscript.

Field-specific reporting

Please select the one below that is the best fit for your research. If you are not sure, read the appropriate sections before making your selection.

[x] Life sciences [ ] Behavioural & social sciences [ ] Ecological, evolutionary & environmental sciences

For a reference copy of the document with all sections, see nature.com/documents/nr-reporting-summary-flat.pdf

Life sciences study design

All studies must disclose on these points even when the disclosure is negative.

Sample size	No statistical tests were used to pre-determine sample sized but the reported sample sizes are similar to what we and others have previously reported (Shen et al., 2021; Chen et al., 2019; Shen et al., 2019; Zhong et al., 2019).
Data exclusions	For optogenetic, chemogenetic, and imaging data, animals were excluded if there were improper viral expression or implantation sites upon post-hoc inspection. Additionally, for behavioral experiments animals were excluded if their behavioral training did not meet a pre-established performance threshold. No other animals were excluded for this study.
Replication	All details provided for methods, reagents, and data analysis were provided to ensure study repeatability. All sample sizes are reported in the main text, figure legends, and supplementary figure legends.
Randomization	Animals are randomly selected as experimental or control groups from the same litters. Auditory stimulation trials were drawn randomly to simulate high or low frequencies and presented to mice in a random order.
Blinding	This study did not require allocation of animals to separate groups. All behavioral and imaging data were collected using an automated fashion, and comparisons are between different trials or sessions for the same animal.

Reporting for specific materials, systems and methods

We require information from authors about some types of materials, experimental systems and methods used in many studies. Here, indicate whether each material, system or method listed is relevant to your study. If you are not sure if a list item applies to your research, read the appropriate section before selecting a response.

Materials & experimental systems

n/a	Involved in the study
☐	☒ Antibodies
☒	☐ Eukaryotic cell lines
☒	☐ Palaeontology and archaeology
☐	☒ Animals and other organisms
☒	☐ Clinical data
☒	☐ Dual use research of concern

Methods

n/a	Involved in the study
☒	☐ ChIP-seq
☒	☐ Flow cytometry
☒	☐ MRI-based neuroimaging

Antibodies

Antibodies used	The following primary antibodies and dilutions were used: Rabbit anti-TH (1:1000; Abeam, ab112), mouse anti-TH (1:1000; Millipore MAB5280), mouse anti-DARPP-32 (1:1000; Santa Cruz Biotechnology sc-271111), goat anti-GFP (1:1000; Rockland, 600-101-215), and Rabbit anti-RFP (1:1000; Rockland, 600-401-379). The following secondary antibodies were used: Donkey anti-rabbit 647 (1:1000; Jackson ImmunoResearch, AB_2492288), Donkey anti-rabbit 594 (1:1000; Jackson ImmunoResearch, AB_2340621), Donkey anti-mouse 594 (1:1000; Jackson ImmunoResearch, AB_2340854), Donkey anti-mouse 647 (1:1000; Jackson ImmunoResearch, AB_2340863), and Donkey anti-goat 488 (1:1000, Thermofisher, A-11055)
Validation	All primary and secondary antibodies were validated in prior studies by our lab and others (2021 Liu et al., 2020 Wang et al., 2019 Chen et al., 2019 Shen et al.). For dopamine neuron identification, commonly used antibodies we used to label Tyrosine hydroxylase (2016 Parker et al.; 2018 da Silva et al.). For medium spiny neuron identification, commonly used labeling of DARPP-32 was used (Gangarossa et al., 2013; Matamales et al., 2020).

Animals and other research organisms

Policy information about studies involving animals; ARRIVE guidelines recommended for reporting animal research, and Sex and Gender in Research

Laboratory animals	C57BL/6J (The Jackson Laboratory), DAT-IRES-Cre (The Jackson Laboratory, 006660), Ai14 (The Jackson Laboratory, 007914), DlR-Cre (The Jackson Laboratory, 37156), A2a-Cre (MMRRC, 036158 UCD) and D2-eGFP/rpl10a (The Jackson Laboratory, 030255) mice (2-4 months old) were used for this study. Mice were housed with free access to food but were water-restricted after the start of behavioral training. Animals were housed in a 12-hour light/dark cycle, and all behavioral experiments were conducted during the animal's dark cycle.
Wild animals	This study did not involve wild animals.
Reporting on sex	Both male and female mice from each strains were used in this study. Due to the limited availability, we did not separate the data from each sex in the analyses in this study.
Field-collected samples	This study did not involve Field-Collected samples
Ethics oversight	All animal procedures were approved by the Stony Brook University Animal Care and Use Committee and carried out in accordance with National Institutes of Health standards.

Note that full information on the approval of the study protocol must also be provided in the manuscript.
